# Author Correction: Direct measurement of superdiffusive energy transport in disordered granular chains

**DOI:** 10.1038/s41467-018-05576-9

**Published:** 2018-07-27

**Authors:** Eunho Kim, Alejandro J. Martínez, Sean E. Phenisee, P. G. Kevrekidis, Mason A. Porter, Jinkyu Yang

**Affiliations:** 1 Department of Aeronautics and Astronautics, University of Washington, Seattle, WA 98195-2400 USA; 2 Division of Mechanical System Engineering & Automotive Hi-Technology Research Center, Chonbuk National University, 567 Baekje-daero, Deokjin-gu, Jeonju-si, Jeollabuk-do, 54896 Republic of Korea; 3 Oxford Centre for Industrial and Applied Mathematics, Mathematical Institute, University of Oxford, Oxford, OX2 6GG UK; 4 Department of Mathematics and Statistics, University of Massachusetts, Amherst, 01003-4515 MA USA; 5 Department of Mathematics, University of California, Los Angeles, 90095 CA USA; 6 CABDyN Complexity Centre, University of Oxford, Oxford, OX1 1HP UK

Correction to: *Nature Communications*; 10.1038/s41467-018-03015-3; published online 13 February 2018

The original version of this Article contained an error in second sentence of the Acknowledgements, which incorrectly read ‘J.Y. and P.G.K. also acknowledge support from US-ARO (W911NF-15-1-0604) and US-AFOSR (FA9550-17-1-011), and P.G.K. also gratefully acknowledges support from the Stavros Niarchos Foundation via the Greek Diaspora Fellowship Program’. The correct version states ‘US-AFOSR (FA9550-17-1-0114)’ in place of ‘US-AFOSR (FA9550-17-1-011)’. This has been corrected in both the PDF and HTML versions of the Article.

