# Supplementary material for: Direct measurement of superdiffusive energy transport in disordered granular chains
Source: Nat Commun. 2018 Feb 13;9:640. doi: 10.1038/s41467-018-03015-3 (PMC5811443; doi:10.1038/s41467-018-03015-3)
Supplement: Supplementary file 1 — Supplementary Information [file 41467_2018_3015_MOESM1_ESM.pdf]

SUPPLEMENTARY FIGURES

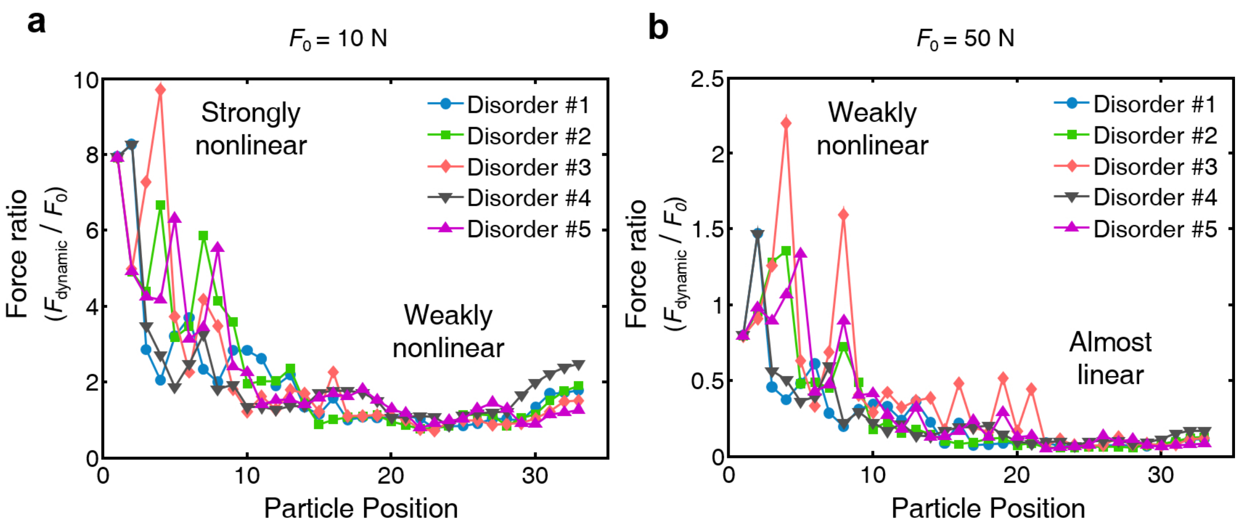

Supplementary Figure 1. **Ratio of maximum dynamic force to static precompression.** (**a**) Ratio of the maximum dynamic force (defined as $F_{\text{dynamic}} = \max(|F - F_0|)$, where F is the propagating wave's force amplitude) to the static precompression F_0 in disordered chains for $F_0 =$ 10 N . (**b**) Ratio of maximum dynamic force to the static precompression in disordered chains for $F_0 = 50$ N.

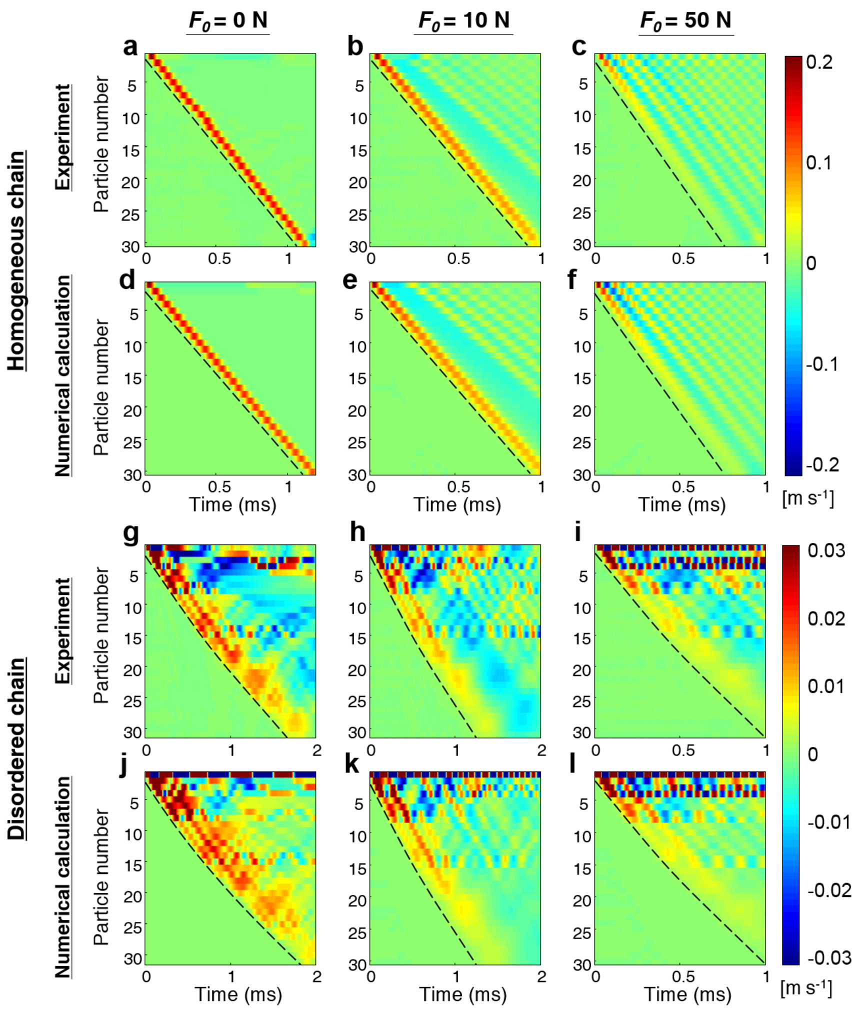

Supplementary Figure 2. **Wave propagation in homogeneous and disordered chains.** (**a**–**f**) Spatiotemporal distributions of particle velocities in a homogeneous chain consisting of aluminum particles with static precompressions of (**a**,**d**) 0 N, (**b**,**e**) 10 N, and (c,f) 50 N. We compare the experimental data (panels **a**–**c**) to numerical simulations (panels **d**–**f**). (**g**–**l**) Spatiotemporal distributions of particle velocities in a disordered chain (the 'Chain 1' configuration in Supplementary Table I) with static precompressions of (**g**,**j**) 0 N, (**h**,**k**) 10 N, and (**i**,**l**) 50 N. We compare the experimental data (panels **g**–**i**) to numerical calculations (panels **j**–**l**). We show dashed arcs to visually track the edges of the velocity distributions.

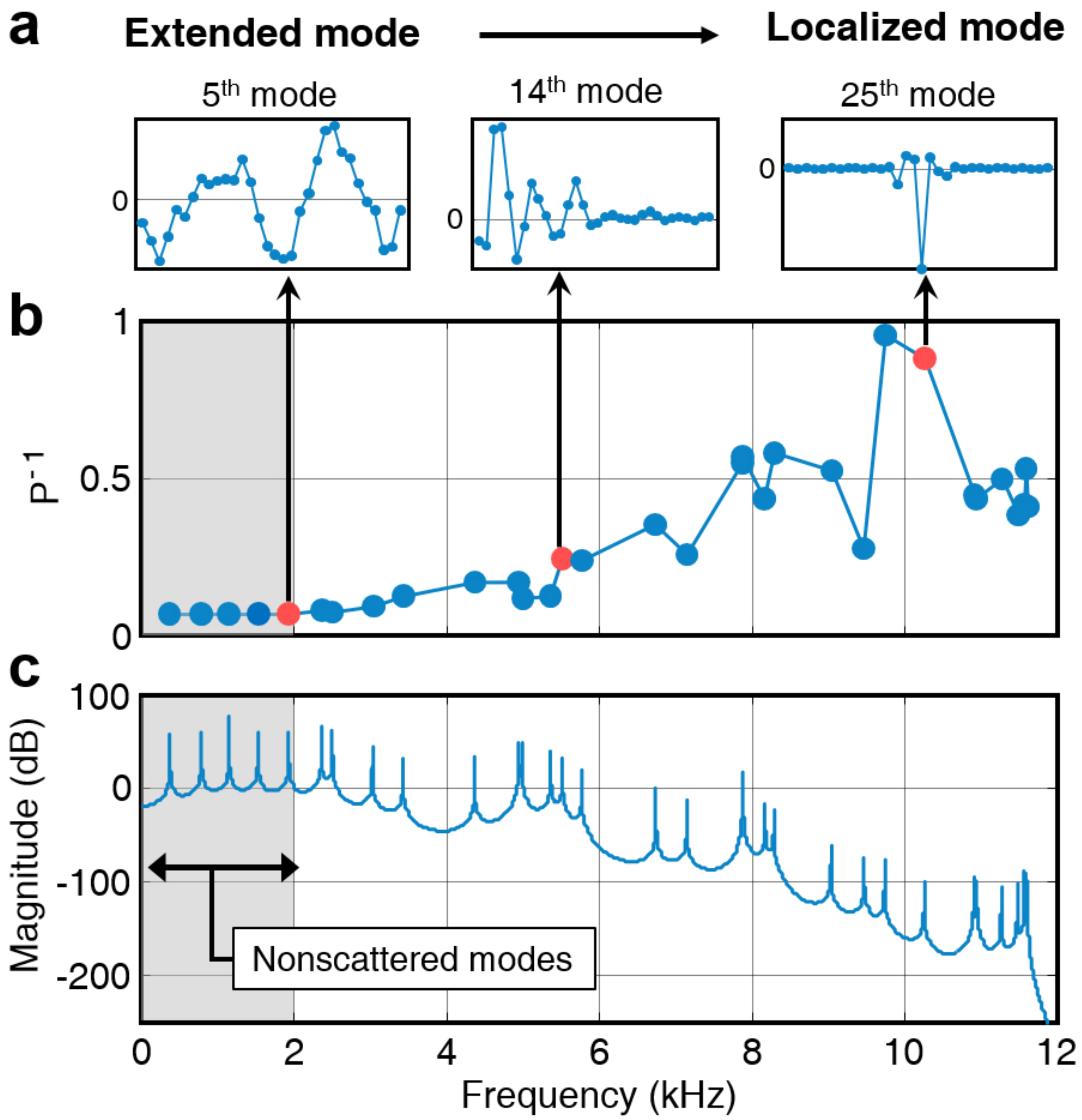

Supplementary Figure 3. **Vibration modes of a disordered chain (Chain 1 from Supplementary Table I) for a static precompression of $F_0 = 50$ N.** (**a**) Mode profiles of the 5th, 14th, and 25th modes. (**b**) Inverse participation ratio (IPR) P^{-1} calculated based on the displacement amplitude for each mode. (**c**) Frequency response function of the disordered chain. The shaded area shows the nonscattered modes (from the 1st to the 5th modes).

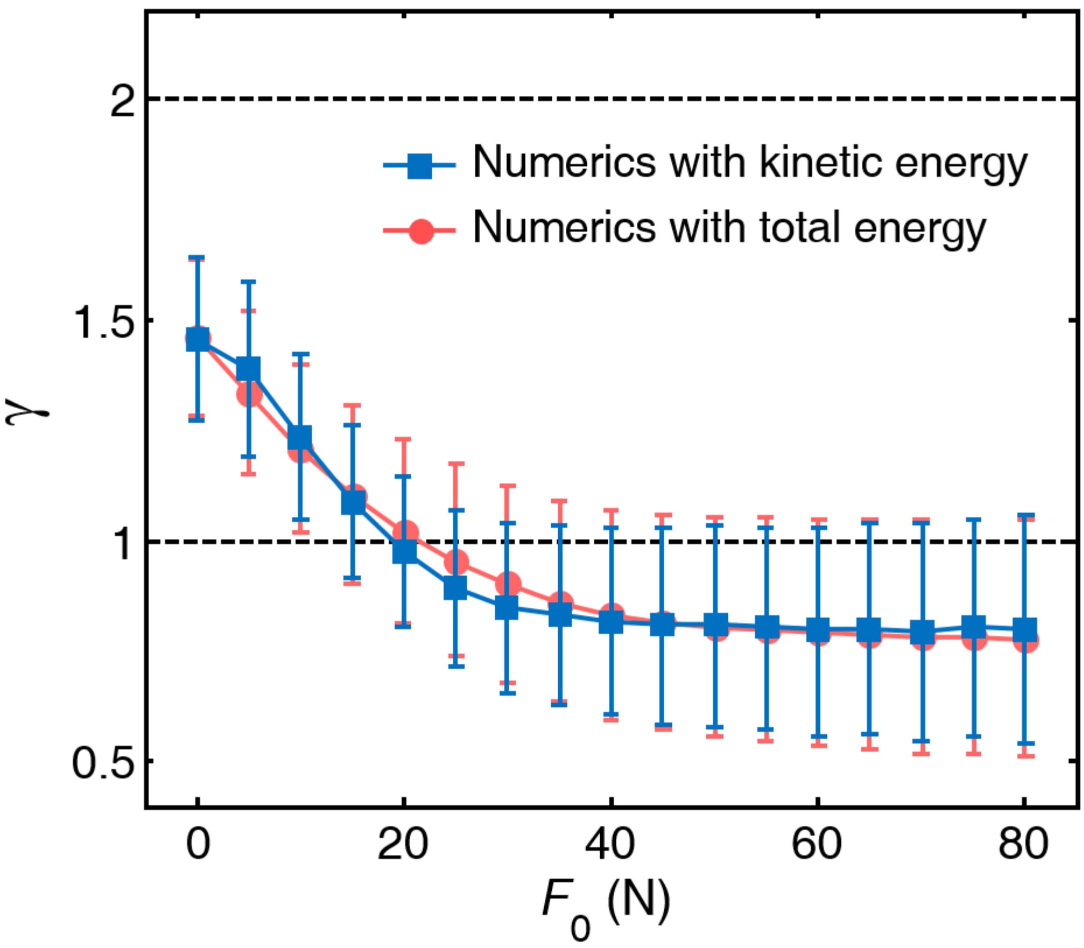

Supplementary Figure 4. **Comparison of second moments from kinetic energy versus those from total energy for different precompression strengths in our numerical simulations of disordered chains.** The blue squares represent calculations based on kinetic energy, and the red circles represent calculations based on total energy (i.e., kinetic energy plus potential energy). The vertical axis gives the exponent γ of the second moment, and the horizontal axis gives the static precompression strength. For these simulations, we use 32-particle chains and a velocity excitation.

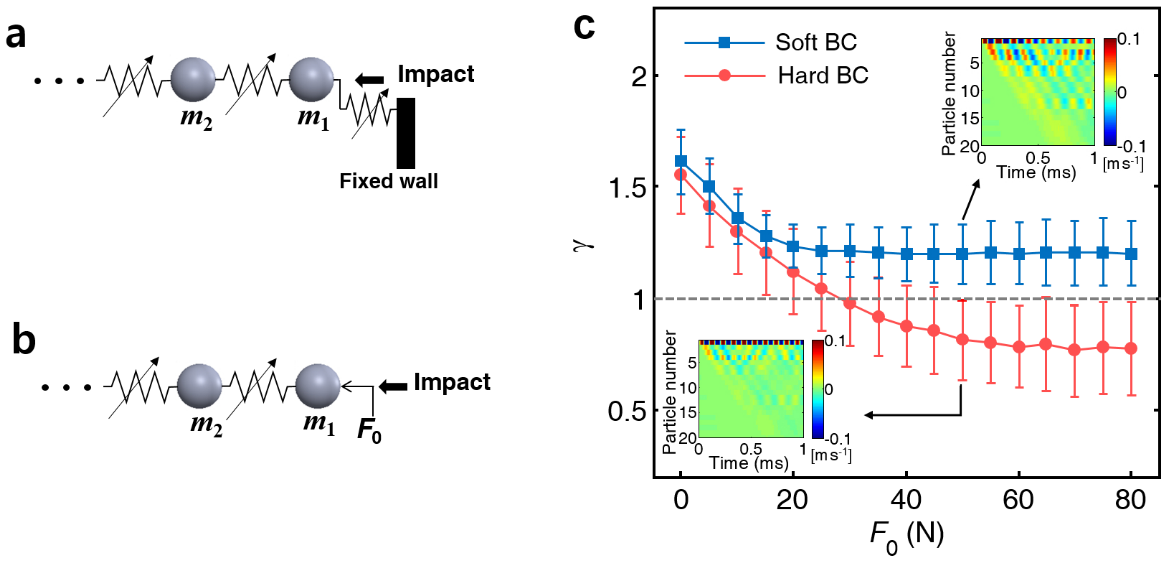

Supplementary Figure 5. **Boundary conditions at the location of the excitation and their corresponding effects on energy transport.** (**a**) Schematic of an excitation as a hard boundary constrained by a fixed wall. (**b**) Schematic of an excitation as a soft boundary (specifically, a free boundary) in which precompression is applied directly to the first particle without any constraints. In both panels, the depicted springs represent Hertzian interactions. (**c**) Comparison of the second moment m_2 of the kinetic energy, averaged over 100 realizations, for granular chains with two different boundary conditions at the right end: a hard boundary (red circles) and soft boundary (blue squares). The insets represent spatiotemporal distributions of the propagation velocity for particles in chains with the two different boundary conditions.

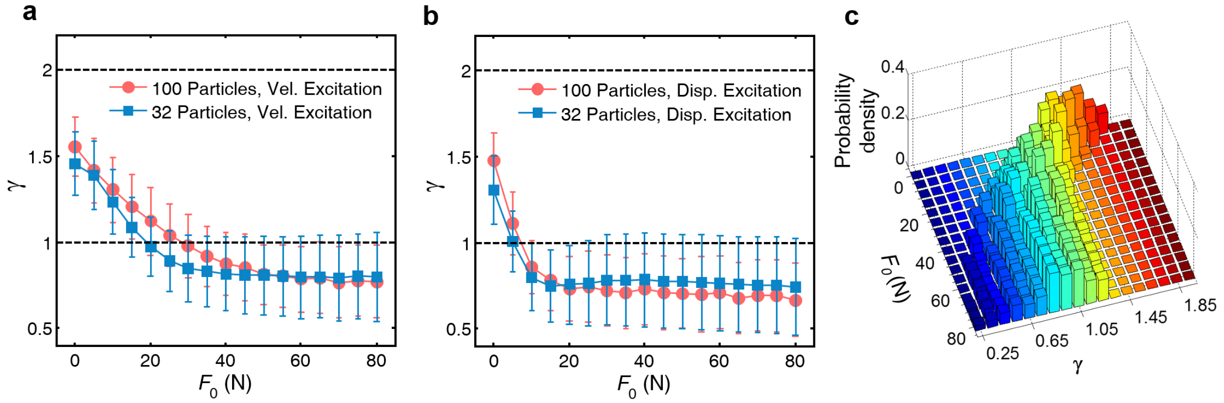

Supplementary Figure 6. **Comparison of exponents γ of the second moment m_2 of the kinetic energy in short versus long chains.** We compare the exponents between short chains (32 particles) and long chains (100 particles) for (**a**) a velocity excitation and (**b**) a displacement excitation. We use 100 chains for each case, and we generate them randomly by independently choosing each particle in the chain as either tungsten-carbide and aluminum particles with equal probability. (**c**) Probability density as a function of statistic precompression F_0 and spreading exponent γ.

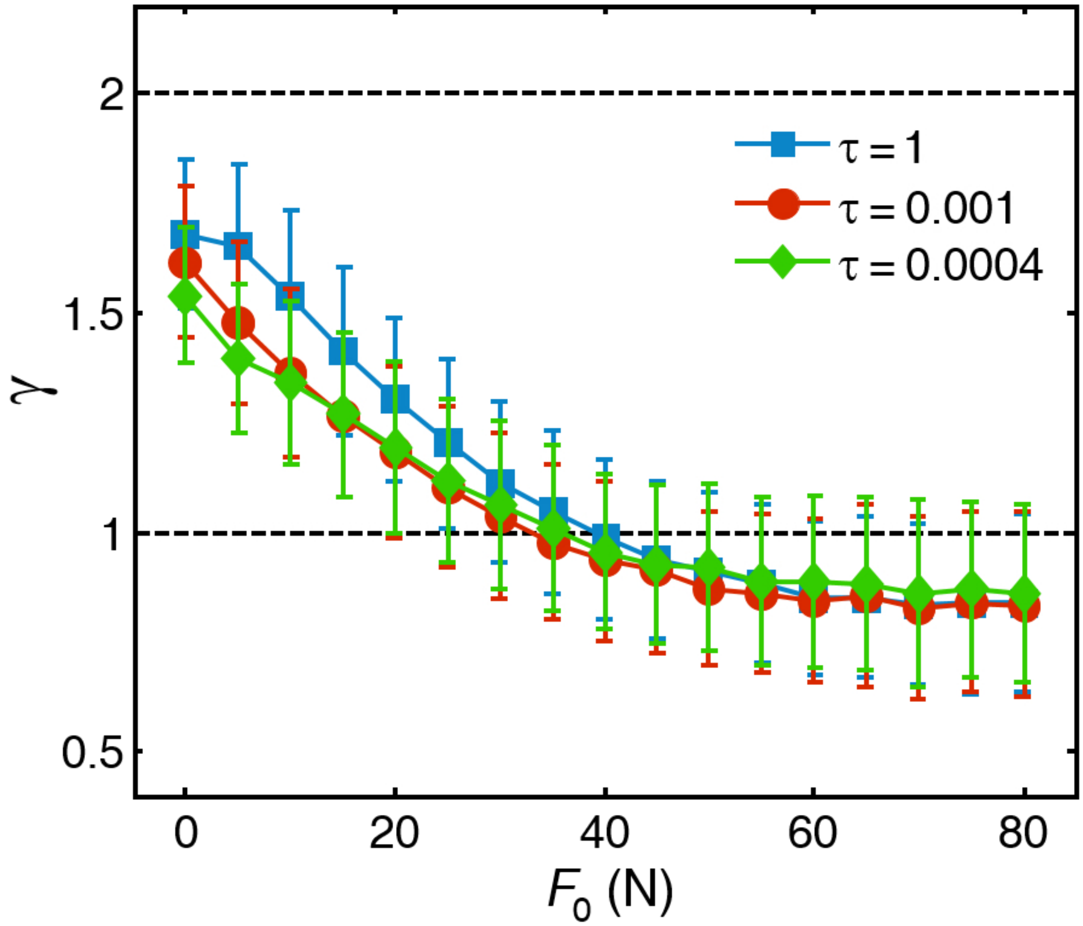

Supplementary Figure 7. **Effect of dissipation on energy spreading in disordered chains.** Exponent γ of the second moment m_2 of kinetic energy for various dissipation coefficients $(1/\tau)$ as a function of precompression strength. We show means of numerical simulations from 100 disordered chains with 100 particles each over the time period $[0.1, 3]$ ms.

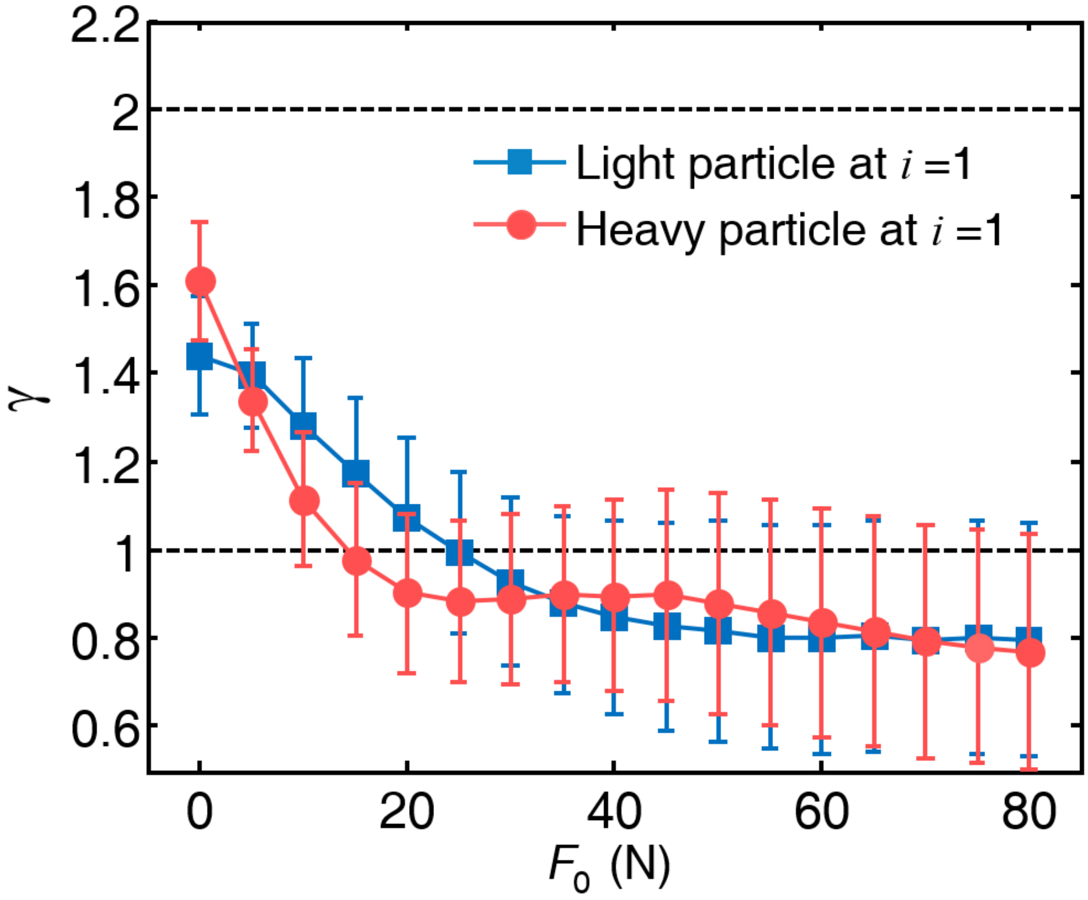

Supplementary Figure 8. **Effect of the choice of material of the first particle on energy spreading.** We compare the exponent γ of the second moment m_2 of the kinetic energy between chains that have a light particle (aluminum) at $i = 1$ (i.e., in the first position) and chains that have a heavy particle (tungsten-carbide) at $i = 1$. All chains have 100 particles and a fixed-wall boundary at the right.

SUPPLEMENTARY TABLE

Supplementary Table I. Configurations of the five disordered chains in our experiments.

Disordered chain	Order of particles (1: Tungsten-Carbide; 2: Aluminum)
Chain 1	[2, 1, 2, 2, 1, 1, 2, 2, 1, 1, 1, 1, 1, 2, 2, 1, 2, 1, 2, 2, 1, 1, 2, 2, 2, 1, 2, 1, 1, 1, 2, 1]
Chain 2	[2, 2, 2, 1, 2, 1, 1, 1, 1, 2, 1, 2, 1, 1, 2, 2, 2, 1, 1, 2, 1, 2, 2, 1, 2, 2, 1, 1, 2, 1, 1, 1]
Chain 3	[2, 2, 1, 1, 2, 2, 1, 1, 2, 2, 2, 2, 2, 2, 2, 1, 2, 1, 1, 1, 1, 2, 1, 2, 2, 1, 1, 2, 1, 1, 1, 2]
Chain 4	[2, 1, 2, 2, 2, 2, 1, 2, 1, 2, 2, 1, 2, 1, 2, 1, 1, 1, 1, 1, 2, 2, 1, 1, 2, 2, 1, 1, 1, 1, 1, 1]
Chain 5	[2, 2, 2, 2, 1, 2, 1, 1, 2, 1, 2, 2, 1, 2, 2, 1, 1, 1, 1, 2, 1, 2, 2, 2, 1, 1, 1, 1, 2, 1, 2, 1]

SUPPLEMENTARY NOTE 1: NUMERICAL AND EXPERIMENTAL RESULTS FROM HOMOGENEOUS AND DISORDERED GRANULAR CHAINS, AND THE DETAILED CONFIGURATIONS OF THE DISORDERED CHAINS.

In Supplementary Figure 2, we show the full-field particle velocities for a homogeneous chain that consists of aluminum particles only (top two rows) and a disordered chain composed of aluminum and tungsten-carbide particles (bottom two rows) in strongly nonlinear, weakly nonlinear, and almost linear scenarios (in the left, middle, and right panels, respectively). In each group, the upper and lower rows show our experimental and numerical results, respectively. Our numerical results corroborate our experimental measurements.

In Supplementary Figure 2 and also in Figure 2 of the main manuscript, we report disordered-chain results for the first configuration ('Chain 1') of the 32-particle disordered chains from Supplementary Table I. We assemble each disordered chain by randomly choosing each individual particle (except for the first one) in the chain as either aluminum or tungsten-carbide with equal probability. This splits the statistical ensemble into two sub-ensembles: (i) chains with the first particle fixed as aluminum and (ii) chains with the first particle fixed as tungsten-carbide. We intentionally choose to examine case (i), because it facilitates transfer of mechanical energy from the striker particle to the chain. This observation is based on preliminary experimental observations using both sub-ensembles. In this case, our experiments have mean energy transfer rates from the striker to the chain of 75.5%, 76.1%, and 85.8% for 0 N, 10 N, and 50 N precompression, respectively. Although we focus our experimental efforts on studying case (i), we show numerically that case (ii) has similar transport properties as case (i). For instance, in both cases, we observe a transition from subdiffusive to superdiffusive behavior, although the precise values for the energy spreading-rate exponents γ differ. (See Supplementary Note 11 for more details.)

SUPPLEMENTARY NOTE 2: NONLINEARITY OF STRESS WAVES PROPAGATING IN DISORDERED CHAINS.

In a disordered granular chain, stress waves scatter as they propagate along the chain, which in turn results in a diminution of the wave amplitude. When one applies precom-

pression to a chain, this amplitude decrease changes the nonlinearity strength in the system as the stress waves propagate along the chain. One can estimate the nonlinearity strength of the propagating wave based on the ratio of its dynamic force to the static precompression. We use the following qualitative criterion for characterizing regimes with different nonlinearity strengths. We say that dynamics is 'strongly nonlinear' when the force ratio $F_{\text{dynamic}}/F_0 \gg 1$, 'weakly nonlinear' when $F_{\text{dynamic}}/F_0 \approx 1$, and 'almost linear' (which is also known as 'nearly linear', or sometimes just 'linear' as a shorthand) when $F_{\text{dynamic}}/F_0 \ll 1$. The maximum dynamic force is $F_{\text{dynamic}} = \max(|F - F_0|)$, where F is the propagating wave's force amplitude and F_0 is the static precompression. In Supplementary Figure 1a, we show F_{dynamic}/F_0 when $F_0 = 10$ N. Initially, a strongly nonlinear wave forms, but it subsequently becomes a weakly nonlinear one due to scattering and localization. In Supplementary Figure 1b, we show the force ratio for $F_0 = 50$ N precompression, where a weakly nonlinear wave becomes an almost linear one as it propagates.

SUPPLEMENTARY NOTE 3: LINEAR MODES OF A DISORDERED CHAIN AND ITS FREQUENCY RESPONSE FUNCTION.

When precompression is strong enough compared to the dynamic force, one can linearize Equation (3) in the main text. Using this linearization, one obtains an approximate description of a granular chain's dynamics as a superposition of the temporal evolution of different vibration modes. The weight of each mode depends on the initial excitation. For a disordered chain, vibration modes with predominantly low frequencies are delocalized, producing what are sometimes called 'nonscattered modes'[1]. The number q_{ns} of nonscattered modes satisfies $q_{\text{ns}} \sim \sqrt{N}$ as $N \to \infty$, where N is the total number of particles in the chain. In our 32-particle chains, roughly the first six modes are nonscattered modes. Consequently, the waves with frequencies associated with nonscattered modes tend to propagate along the chain without localization. For progressively higher frequencies, vibration modes become localized, reducing the effective number of particles that oscillate with an amplitude that differs substantially from 0. We quantify this phenomenon by computing the inverse participation ratio (IPR) P^{-1}, which we show in Supplementary Figure 3. We also show representative vibration modes and frequency response functions of a disordered chain (using Chain 1 from Supplementary Table I) at $F_0 = 50$ N. Because the IPR represents the degree

of localization, an extended mode — corresponding to a low-frequency wave mode with a long wavelength — has a low value of P^{-1}, whereas a localized mode has P^{-1} close to 1.

SUPPLEMENTARY NOTE 4: EXPONENTIAL FIT OF KINETIC-ENERGY DECAY IN THE ANDERSON-LIKE MODE.

To support the exponential fit that we use to characterize the early decay of the kinetic-energy distribution (see Figure 3 in the main manuscript), we perform an Anderson–Darling test[2]. Because we estimate the parameter of the exponential trend from data, to implement the test, we apply a Monte-Carlo procedure with 1000 data sets generated under the null hypothesis of the exponential distribution $\bar{K}_i \propto e^{-0.54i}$. Using particles $i \in \{2, \ldots, 10\}$, we obtain a p-value of 0.96, so it passes the standard statistical test indicating that there is no significant departure from normality.

SUPPLEMENTARY NOTE 5: COMPARISON OF SPREADING EXPONENT γ BETWEEN KINETIC ENERGY AND TOTAL ENERGY.

In Supplementary Figure 4, we compare the exponents γ of the second moment ($m_2(t) \sim t^\gamma$) of the kinetic energy with those that we calculate using the total energy in our numerical simulations of 32-particle disordered chains with a velocity excitation (see Supplementary Note 10). This figure illustrates that, despite the observed oscillations of $m_2(t)$ because of the kinetic–potential energy exchange in the propagating waves, the exponents of the experimentally measurable kinetic energy are essentially the same as those obtained from total energy.

SUPPLEMENTARY NOTE 6: BOUNDARY EFFECTS

Previous studies reported that when the disorder is uncorrelated (as in our case), velocity excitations in the bulk exhibit superdiffusive transport regardless of the nonlinear regime, whereas displacement excitations transition from subdiffusive transport in the almost linear regime to superdiffusive spreading in the strongly nonlinear regime[3,4]. Interestingly, when we excite the first particle at the fixed boundary of a granular chain, we observe that both velocity and displacement excitations have a transition from subdiffusive to superdiffusive

transport (see Supplementary Figure 6). To investigate the effect of boundary conditions on energy-transport mechanisms, we conduct numerical simulations using 100-particle chains and compare two scenarios. One scenario is a hard boundary fixed with a massive wall (as in the experimental setup), and the other is a soft boundary in which we apply a consistent precompression force to the first particle (see Supplementary Figures 5a,b). For a fixed boundary with a wall, the boundary particle ($i = 1$) interacts with three objects: the striker ($i = 0$), an adjacent particle ($i = 2$), and the wall ($i = w$). Its equation of motion is

$$
\begin{aligned}
m_1 \frac{d^2 u_1}{dt^2} &= A_{0,1} \left[\delta_{0,1} + u_0 - u_1\right]_+^{3/2} - A_{1,2} \left[\delta_{1,2} + u_1 - u_2\right]_+^{3/2} \\
&\quad + A_{w,1} \left[\delta_{w,1} - u_1\right]_+^{3/2} - \frac{m_1}{\tau} \frac{du_1}{dt} \, ,
\end{aligned}
\tag{1}
$$

where first and second terms on the right-hand side represent, respectively, the interactions of the first particle with the striker and the second particle. The third term on the right-hand side models the interaction with the fixed wall, and the last term represents dissipation in the first particle. For a free boundary with a constant force, the third term in Supplementary Equation (1), which represents the interaction with the wall, is replaced with a constant force F_0. This yields the equation

$$
\begin{aligned}
m_1 \frac{d^2 u_1}{dt^2} &= A_{0,1} \left[\delta_{0,1} + u_0 - u_1\right]_+^{3/2} - A_{1,2} \left[\delta_{1,2} + u_1 - u_2\right]_+^{3/2} \\
&\quad + F_0 - \frac{m_1}{\tau} \frac{du_1}{dt} \, .
\end{aligned}
\tag{2}
$$

One can construe such a free boundary as an extreme case of a soft boundary.

We conduct numerical simulations using both boundary conditions. In Supplementary Figure 5c, we observe that the choice of boundary condition has a negligible effect for weak precompression, and the energy spreading exhibits similar superdiffusive behavior for both hard and soft boundaries. However, for progressively stronger precompression, the disparity between the two scenarios becomes larger. For hard boundaries, a significant amount of excitation energy is trapped near the boundary for a long time (compare the insets of Supplementary Figure 5c), so the exponent γ of m_2 suggests a trend towards subdiffusive spreading as one increases precompression. In contrast, for a soft boundary, localization is weaker and the energy spreads more rapidly. The resulting mean exponent γ asymptotically approaches a particular value (roughly 1.2) in the superdiffusive regime. This asymptotic value depends on the mass ratio of the heavy and light particles, similar to what has been

described for the bulk of a granular chain[4], and also on the initial excitation. Note that we estimate values of γ using a specified finite duration both in our numerical computations and in our experiments, so one can expect some discrepancies between these values and ones that are calculated over an infinite time horizon (i.e., asymptotic values as $t \to \infty$). However, in past work[3], it has been observed in numerical computations that the value of γ in an initial time period (of about 0.1–1 ms) seems to persist for a long time without significant changes.

Finally, comparing the results from Supplementary Figure 5c with those from Figure 5 of the main manuscript, we deduce that the boundary condition of our experimental setup corresponds to the hard boundary condition (i.e., the setup shown in Supplementary Figure 5a), based on the fact that $\gamma \leq 1$. This makes sense, given the clamped configuration of the first particle in the chain. (See the inset of Figure 1 of the main manuscript.)

SUPPLEMENTARY NOTE 7: COMPARISON OF M_2 BETWEEN SHORT AND LONG CHAINS.

We use numerical simulations to compare the exponent γ of the second moment m_2 of the kinetic energy between short chains (32 particles) and long chains (100 particles). We do this comparison for both velocity and displacement excitations. Previous studies reported that γ achieves a specific value[1,3,4] faster in time with velocity excitations than with displacement excitations[3,4]. In our simulations, we observe that the variation of the exponents is relatively small after about 0.1 ms, similar to the trend reported in 3. Therefore, we estimate the exponents during the time window $[0.1, 1]$ ms for the short chains and during the window $[0.1, 3]$ ms for the long chains using a least-squares fit. As we show in Supplementary Figures 6a (velocity excitations) and 6b (displacement excitations), the results from the short chains have minor discrepancies from those for the long chains, especially when one considers the large standard deviation (see the error bars) of the extracted exponents. In Supplementary Figure 6c, we show the statistical distribution of the exponents calculated using 100 chains of 32 particles for a velocity excitation (corresponding to the curve with blue squares in Supplementary Figure 6a). For weak precompression, the long chains have relatively large exponents for both velocity and displacement excitations. We expect that this observation is related to the detrapping of localized energy in the weakly nonlinear regime[4]. We also observe that the exponents for the displacement excitations are

smaller than corresponding ones for the velocity excitation. (Compare Supplementary Figures 6a and 6b.) This arises because a displacement excitation includes broadly-distributed frequencies, whereas a velocity excitation includes mostly low-frequency signals.

SUPPLEMENTARY NOTE 8: EFFECT OF DISSIPATION ON ENERGY SPREADING.

We investigate the effect of dissipation on energy spreading. For simplicity, we use linear damping, as indicated in the last term in Equation (3) of the main text. This is a common choice for incorporating dissipative effects in models of granular chains[3]. However, we note in passing that numerous models have been proposed to capture dissipative effects, and the issue of deriving a proper qualitative and quantitative incorporation of dissipation is an open problem[5]. In Supplementary Figure 7, we show the effect of the dissipation time scale τ on the exponent γ of the second moment m_2 of kinetic energy when we vary the static precompression F_0. When the effect of damping is significant ($\tau = 0.0004$), γ decreases slightly in the weakly and strongly nonlinear regimes. (Compare the blue squares with the red circles and green diamonds in Supplementary Figure 7.) However, we find that the global dissipation term does not significantly change the characteristics of energy spreading.

SUPPLEMENTARY NOTE 9: TESTING FOR PLASTICITY.

We also test for the possibility of plastic effects. First, we note that we did not observe any visible dents on the surface of the aluminum beads after repeated experiments. Additionally, the test data sets do not exhibit significant deviations from each other after repeated tests. To examine this possibility further, we numerically calculate (results not shown) the von Mises stress of the particles in contact. We find that although plastic deformation may happen inside the aluminum particles, it would have only a minor effect on the particle surfaces.

SUPPLEMENTARY NOTE 10: TYPES OF INITIAL CONDITIONS: VELOCITY AND DISPLACEMENT EXCITATIONS

We consider two types of initial conditions in our numerical simulations. Similar to simulations in prior work[3,4] for the bulk of a granular chain, in our examination of boundary-induced excitations, we conduct simulations with two types of initial conditions. We apply a velocity excitation that consists of an initial perturbation of the velocity of a single particle, with all other particles starting with zero velocity and all particles starting with zero displacement (see Supplementary Figure 6a). We also consider a displacement excitation of the position from equilibrium of a single particle (see Supplementary Figure 6b). In Supplementary Note 7, we used both types of initial conditions in our comparisons of short and long chains.

SUPPLEMENTARY NOTE 11: EFFECT OF THE MATERIAL OF THE FIRST PARTICLE IN A GRANULAR CHAIN ON ENERGY SPREADING.

We compare the exponents γ of the second moment m_2 of the kinetic energy for two types of chains: one with a light particle (aluminum particle) in the first ($i = 1$) spot and the other with a heavy particle (tungsten-carbide) in that spot. In Supplementary Figure 8, we show computational results for these two cases from numerical simulations of 100 chains with 100 particles each. For weak precompression, the two chains exhibit very different behaviors. However, as we consider progressively stronger precompression, the exponents of the two sets of chains approach the same value, and the energy in both sets is transported in a subdiffusive way. In the main text, we used light (i.e., aluminum) particles at the $i = 1$ spot to facilitate the transport of energy to the granular chain from the striker particle.

SUPPLEMENTARY REFERENCES

[1]P. K. Datta and K. Kundu, Energy transport in one-dimensional harmonic chains, Phys. Rev. B **51**, 6287–6295 (1995).

[2]T. W. Anderson and A. D. Darling, Asymptotic theory of certain "goodness-of-fit" criteria based on stochastic processes, Ann. Math. Stats. **23**, 193–212 (1952).

[3]A. J. Martínez, P. G. Kevrekidis, and M. A. Porter, Superdiffusive transport and energy localization in disordered granular crystals, Phys. Rev. E **93**, 022902 (2016).

[4]V. Achilleos, G. Theocharis, and Ch. Skokos, Energy transport in one-dimensional disordered granular solids, Phys. Rev. E **93**, 022903 (2016).

[5]C. Chong, M. A. Porter, P. G. Kevrekidis, and C. Daraio, Nonlinear coherent structures in granular crystals, J. Phys. Cond. Matt. **29**, 413003 (2017).
